# A case of hypoglycemic hemiparesis and literature review

**DOI:** 10.3109/03009734.2011.652748

**Published:** 2012-08

**Authors:** Tetsuhiro Yoshino, Shu Meguro, Yukie Soeda, Arata Itoh, Toshihide Kawai, Hiroshi Itoh

**Affiliations:** Division of Endocrinology, Metabolism and Nephrology Department of Internal Medicine Keio University, School of Medicine, Shinjuku, Japan

**Keywords:** Diabetes, glimepiride, hypoglycemic hemiparesis, magnetic resonance, metformin

## Abstract

An 89-year-old man with diabetes treated with metformin 500 mg/day and glimepiride 4 mg/day was hospitalized because of hypoglycemic right hemiparesis and dysarthria (casual glucose value 1.8 mmol/L), which resolved quickly following administration of 40 mL of 40% dextrose. Hemiparesis is a rare symptom (4.2%) of hypoglycemia. There are about 200 case reports of hypoglycemic hemiparesis. The average glucose level at which hemiparesis developed was 1.8 mmol/L. Right-sided hemiparesis predominated (R 66%; L 34%). On imaging studies, abnormal findings were frequently observed in the internal capsule or splenium of the corpus callosum. The mechanism of hemiparesis is not fully understood. The existence of cases in which hypoglycemia cannot be distinguished from stroke on imaging studies suggests the importance of measurement of the blood glucose level when the symptoms of stroke are first recognized.

## Introduction

Lawrence Tierney stated: ‘A stroke is never a stroke until it has received 50 of D50’. This maxim highlights hypoglycemic hemiparesis and means that hypoglycemia should not be forgotten when treating a patient who is suspected to have had a stroke.

According to one retrospective chart review of 59 patients, the most common neuroglycopenic symptoms are confusion (83%) and personality change (64%), while the most common autonomic complaints are diaphoresis (69%) and tremor (24%) (1).

Hemiparesis is a rare sign of hypoglycemia (2–4) and is often overlooked. In such cases, unwarranted investigations (such as head computed tomographic scan) and treatment (such as oral aspirin) are performed (5,6).

We here present a case of hypoglycemic hemiparesis and a review of the previous literature.

## Case

An 89-year-old man with a 20-year history of type 2 diabetes mellitus was hospitalized because of a hypoglycemic attack. He had regularly attended the Department of Geriatric Internal Medicine because of hypertension and dyslipidemia. He had a previous history of cerebral infarction of the right corona radiata, bleeding gastric ulcer, hyperuricemia, chronic kidney disease (stage 3), benign prostatic hypertrophy, and stable angina. His medication included metformin 500 mg/day, glimepiride 4 mg/day, aspirin 100 mg/day, omeprazole 10 mg/day, allopurinol 100 mg/day, pravastatin sodium 10 mg/day, and naftopidil 50 mg/day. He had been smoking half a pack a day for 70 years. On his last regular visit in July 2010, glycated hemoglobin (HbA1c) was 6.9%, and his usual medications were prescribed. In September 2010 he reported that his physical condition had been poor, and he had been eating only a small lunch but taking his regular medications. When he called his family, dysarthria was noticed. Because right hemiparesis developed the next morning, he was brought to our emergency room with his daughter.

On physical examination, he had intact cranial nerves except for dysarthria, a shallow right nasolabial fossa, tongue deviation to the right, and muscle weakness of the right arm with symmetrical, not brisk reflexes. He had no ataxia or numbness, but had decreased vibration sense at both ankles. Body mass index was 21.4, blood pressure was 123/54 mmHg, and heart rate was regular and 64 beats per minute. Body temperature was 36.7°C, and Glasgow coma scale was 14 (E4V4M6). He could say his name but not ‘pen’ or ‘glasses’. There were no other remarkable findings on physical examination including conjunctivae, oral mucosa, cervical lymph nodes, thyroid, heart, respiratory sounds, abdomen, and skin.

Laboratory findings showed hypoglycemia (casual glucose value 1.8 mmol/L), worsening of renal dysfunction (urea nitrogen 12 mmol/L, creatinine 0.22 mmol/L), anemia (hemoglobin 9.8 g/dL, hematocrit 29.2%), hyperkalemia (5.8 mmol/L), and decreased high-density lipoprotein cholesterol (0.62 mmol/L). The rest of the findings were normal, including complete blood cell count, coagulation tests, serum transferases, sodium, calcium, phosphate, and C-reactive protein. Glycated hemoglobin (HbA1c) was 6.9%, and glycated albumin was 21.6% on admission.

Because of the laboratory findings, 40 mL of 40% dextrose solution was given intravenously. Blood glucose level rose to 9.6 mmol/L, his signs resolved quickly, and hypoglycemic hemiparesis was diagnosed. Glimepiride, metformin, and valsartan were discontinued. As the neurological signs disappeared quickly on improvement of blood glucose, it was easy to exclude an ischemic stroke. Emergency head imaging was not done. After 48 hours' observation, he was discharged, and no relapse of hypoglycemic attack has been documented to date. The 24-hour urine collection showed no microalbuminuria, and creatinine clearance was calculated as 29 mL/min. He had no diabetic retinopathy. One month after discharge, head magnetic resonance imaging showed only old multiple cerebral infarcts, which did not differ from the previous imaging findings.

## Glycated hemoglobin

The value of HbA1c (%) is estimated as an NGSP (National Glycohemoglobin Standardization Program) equivalent value (%) calculated by the formula HbA1c (%) = HbA1c (JDS: Japan Diabetes Society) (%) + 0.4%, based on the relational expression of HbA1c (JDS) (%) measured by the previous Japanese standard substance and measurement methods and HbA1c (NGSP) (7).

## Literature search

A search of MEDLINE from January 1950 to December 2010 was conducted, and of ICHUSHI from January 1983 to December 2010. ICHUSHI is an abbreviation for Igaku-Chuo Zasshi (Japanese Central Review of Medicine), the Japanese database for medical journals, produced by the Japan Medical Abstract Society, the largest database of medical articles in Japan, and includes more than 5,000 journals, with many published articles and abstracts of scientific meetings, mainly written in Japanese.

Articles were searched for using the keywords ‘hypoglycemic hemiplegia’, ‘hypoglycemic hemiparesis’, combined with the keyword ‘hypoglycemia’, ‘hemiplegia’, or ‘hemiparesis’. Only the abstract was read if the relevant article was written in a foreign language. We also searched the reference lists of the included studies/articles (Appendices 1–3, can be found online at http://www.informahealthcare.com/doi/abs/10.3109/03009734.2011.652748.).

## Discussion

The present case of hypoglycemic hemiparesis developed in a patient who was receiving treatment for type 2 diabetes mellitus. It is thought that there were two causes of hypoglycemia in this case. First, he took his medications as usual though the amount of food ingested decreased on the days he was sick. Second, there was a delay in the excretion of glymepiride because of decreased renal function due to his age, diabetic nephropathy, and a further decrease due to dehydration. Dehydration was confirmed by history and hemoconcentration in his laboratory data. It is assumed that the risk of hypoglycemia would be increased by delayed glimepiride excretion in an elderly patient with decreased kidney function (8,9).

As for metformin, because of the increased risk of fatal lactic acidosis, it is recommended that it is not used in patients with decreased renal function (10). We discontinued metformin in this patient after this episode, although it is reported that it may be used safely if glomerular filtration rate does not fall below 30 mL/min (11).

In our literature review we found about 200 reported cases of hypoglycemic hemiparesis to date (2–6,12–66) since the two cases reported by Ravid and one case reported by Diecke in 1928 (12,13).

In general, sympathetic nervous symptoms and impaired consciousness are the major symptoms of hypoglycemic attack, and hemiparesis is rare (7 of 168 cases (4.2%)) (2-4). There was no difference based on sex, age, or cause of hypoglycemia. The average blood glucose level at which hemiparesis developed was 1.8 mmol/L (95% confidence interval 1.7–2.0). Although we did not take an electroencephalogram in this case, a previous case report pointed out that almost the same level of blood glucose could be a threshold of electroencephalographic changes during hypoglycemia (67). These facts suggested there was a threshold to maintain the brain functions at this glucose level. Hemiparesis showed a right-sided predominance (right 66%; left 34%), but the reason is unclear. The difference in metabolism or the density of neuronal tissue between the hemispheres might contribute to the increased vulnerability of the right hemisphere to hypoglycemia (68). However, not all the cases may have these differences.

The mechanism of hemiparesis is not well understood either. Vasospasm, failure of blood flow autoregulation, and change in hypoglycemic tolerance are hypothesized (16). Hypoglycemic vasospasm is observed in the coronary artery (69,70). Hypoglycemic cerebral artery spasm could explain the transient changes on imaging studies. A previous case report suggested that hypoglycemia could cause a brain cell injury in the same way as hypoxia or ischemia (71). The mechanism of vasospasm is also unknown.

There are various possible causes of hypoglycemia, including attempted suicide (57), accidental ingestion by infant (39), and insulin shock treatment for schizophrenia (72). Furthermore, there is an overwhelming number of different oral hypoglycemic agents and insulin for the treatment of diabetes.

Twenty-two case reports showed abnormal findings on computed tomography (CT), single-photon emission computed tomography (SPECT), diffusion-weighted images (DWI), or apparent diffusion coefficient (ADC) mapping of magnetic resonance imaging (MRI). An abnormal finding was observed on the contralateral side or both sides of the internal capsule in 13 (65%) of 22 cases, and in the splenium of the corpus callosum in 6 cases (30%). There are some case reports of abnormal findings in the cortical layer, putamen, corona radiata, and pons. In previous reports, the vulnerable portions of the brain in case of hypoglycemic encephalopathy were the cerebral cortex, basal nuclei, substantia nigra, hippocampus, thalamus, or hypothalamus (73,74). In contrast, imaging abnormality was pointed out in the internal capsule or the splenium of the corpus callosum in many case reports of hypoglycemic hemiparesis.

The time from onset to treatment is mentioned as one of the prognostic factors of hypoglycemia (75). The existence of cases in which hypoglycemia cannot be distinguished from stroke on imaging studies suggests the importance of measurement of the blood glucose level when the symptoms of stroke are first recognized.

## Conclusion

We report a case of hypoglycemic hemiparesis. It is an important differential diagnosis of stroke, though hemiparesis is a rare symptom of hypoglycemia. Right-sided hemiparesis predominates, and a transient ischemic focus in a site corresponding to a symptom may be seen on brain imaging. In patients who present with stroke, it is important that the initial treatment regimen must bear in mind the possibility of hypoglycemia.

## Supplementary material available online at

http://www.informahealthcare.com/doi/abs/10.3109/03009734.2011.652748.

Appendix Tables 1–3.
